# Diagnoses after newly recorded abdominal pain in primary care: observational cohort study

**DOI:** 10.3399/BJGP.2021.0709

**Published:** 2022-06-28

**Authors:** Sarah J Price, Niamh Gibson, William T Hamilton, Jennifer Bostock, Elizabeth A Shephard

**Affiliations:** Cancer Diagnosis (DISCO), University of Exeter Medical School, Exeter.; University of Exeter Medical School, Exeter.; Cancer Diagnosis (DISCO), University of Exeter Medical School, Exeter.; Policy Research Unit on Cancer Awareness, Screening and Early Diagnosis, Queen Mary University of London, London.; Cancer Diagnosis (DISCO), University of Exeter Medical School, Exeter.

**Keywords:** abdominal pain, diagnosis, general practice, urinary tract infections, diverticular diseases, primary health care

## Abstract

**Background:**

Non-acute abdominal pain in primary care is diagnostically challenging.

**Aim:**

To quantify the 1-year cumulative incidence of 35 non-malignant diagnoses and nine cancers in adults after newly recorded abdominal pain in primary care.

**Design and setting:**

Observational cohort study of 125 793 Clinical Practice Research Datalink GOLD records.

**Method:**

Participants, aged ≥40 years, had newly recorded abdominal pain between 1 January 2009 and 31 December 2013. Age- and sex-stratified 1-year cumulative incidence by diagnosis is reported.

**Results:**

Most (>70%) participants had no pre-specified diagnoses after newly recorded abdominal pain. Non-malignant diagnoses were most common: upper gastrointestinal problems (gastro-oesophageal reflux disease, hiatus hernia, gastritis, oesophagitis, and gastric/duodenal ulcer) in males and urinary tract infection in females. The incidence of upper gastrointestinal problems plateaued at age ≥60 years (aged 40–59 years: males 4.9%, 95% confidence interval [CI] = 4.6 to 5.1, females 4.0%, 95% CI = 3.8 to 4.2; aged 60–69 years: males 5.8%, 95% CI = 5.4 to 6.2, females 5.4%, 95% CI = 5.1 to 5.8). Urinary tract infection incidence increased with age (aged 40–59 years: females 5.1%, 95% CI = 4.8 to 5.3, males 1.1%, 95% CI = 1.0 to 1.2; aged ≥70 years: females 8.0%, 95% CI = 7.6 to 8.4, males 3.3%, 95% CI = 3.0 to 3.6%). Diverticular disease incidence rose with age, plateauing at 4.2% (95% CI = 3.9 to 4.6) in males aged ≥60 years, increasing to 6.1% (95% CI = 5.8 to 6.4) in females aged ≥70 years. Irritable bowel syndrome incidence was higher in females (aged 40–59 years: 2.9%, 95% CI = 2.7 to 3.1) than males (aged 40–59 years: 2.1%, 95% CI = 1.9 to 2.3), decreasing with age to 1.3% (95% CI = 1.2 to 1.5) in females and 0.6% (95% CI = 0.5 to 0.8) in males aged ≥70 years.

**Conclusion:**

Although abdominal pain commonly remains unexplained, non-malignant diagnosis are more likely than cancer.

## INTRODUCTION

Abdominal pain is the ninth most common reason for consulting primary care, with a prevalence of 2.8%.^1^ The most common diagnoses in patients with abdominal pain in primary care are gastroenteritis, irritable bowel syndrome, urological diseases, and gastritis.^1^ No cause of abdominal pain is identified in approximately one-third of patients, illustrating the diagnostic challenge for family doctors.^1^

The differential diagnoses of abdominal pain are informed by the history and examination. The symptoms can be acute (recent, short duration) or chronic (long-standing or recurrent). A small proportion of patients are clearly seriously unwell, requiring urgent specialist input. Although their diagnosis may be apparent, the severity of their condition drives their management.^2^^–^4 Therefore, the main diagnostic challenge with abdominal pain in primary care is the patient who is not seriously unwell, whose initial investigations and management will be in primary care.

Investigations of patients with abdominal pain fall into three main categories: tests of blood, urine, and faeces; endoscopy; and imaging.^5^ Most laboratory tests are managed wholly within general practice, whereas endoscopy and imaging require secondary care attendance, with the referring doctor retaining clinical responsibility for the patient. Laboratory tests may check for infection (systemic, of urine or stool), anaemia, markers of inflammation, liver function, pregnancy, or for markers of specific diseases, such as coeliac and inflammatory bowel disease.^5^^–^10 Endoscopy is indicated where lower and upper gastrointestinal (GI) cancers are suspected^11^ or to diagnose other conditions, such as diverticular disease^12^ or inflammatory bowel disease.^6^ Other imaging modalities include ultrasound (for example, for suspected ovarian cancer^13^) and computed tomography for abdominal organ pathologies including pancreatic cancer,^11^ renal disease, and diverticular disease.^12^

This study aimed to follow-up a cohort of patients for 1 year after newly recorded abdominal pain in primary care. The 1-year cumulative incidence of pre-specified malignant and non-malignant differential diagnoses of abdominal pain in adults aged ≥40 years was quantified in the expectation that this could assist clinicians in prioritising investigation.

## METHOD

### Study design and setting

This prospective cohort study was undertaken in England using Clinical Practice Research Datalink (CPRD) GOLD primary care data with National Cancer Registration and Analysis Service (NCRAS) linkage.

**Table:** How this fits in

A main diagnostic challenge for GPs is the patient with abdominal pain who is not seriously unwell and for whom the initial investigations and management will be in primary care. This study reports the 1-year cumulative incidence of 35 non-malignant diagnoses and nine intra-abdominal cancers in males and females aged ≥40 years. The most common diagnoses are non-malignant: upper gastrointestinal problems (gastro-oesophageal reflux disease, hiatus hernia, gastritis, oesophagitis, and gastric/duodenal ulcer) in males, and urinary tract infection in females. These results will help inform appropriate testing strategies and the need for specialist referral.

### Sample selection criteria and study size

The CPRD provided data on study participants who had at least one abdominal pain code (see Supplementary Table S1) in their CPRD record between 1 January 2009 and 31 December 2013. The index date was the date of the first abdominal pain code in this period. Participants also had continuous CPRD records meeting up-to-date standards from at least 1 year before the index date and throughout their 1-year follow-up period. Participants were aged ≥40 years on the index date, where age was identified from the CPRD year of birth, assigning a birthday of 1 July. A previous cancer diagnosis is likely to alter primary care consulting behaviour^14^ and cancer suspicion;^15^ therefore, participants with any cancer diagnostic code recorded before the index date were excluded. To ensure ‘newly recorded’ abdominal pain was studied, anyone with an abdominal pain code recorded in the 12 months before the index date was excluded.

The sample sizes were determined for analysis of this dataset in an allied study of abdominal cancer incidence after newly recorded abdominal pain.^16^ The participant numbers provided by the CPRD were powered to give the following margins of error around a 1-year cumulative incidence of 1%:

- aged 40–59 years: 0.1 percentage points (*n* = 29 920 females, *n* = 29 944 males);
- aged 60–69 years: 0.2 percentage points (*n* = 14 955 females, *n* = 14 506 males); and
- aged ≥70 years: 0.1 percentage points (*n* = 23 008 females) and 0.2 percentage points (*n* = 13 460 males).

### Follow-up

NCRAS and CPRD medical records in the year after the index date were searched for pre-specified diagnostic codes (see Supplementary Table S2). Intra-abdominal malignancies were included, plus benign diagnoses presenting with abdominal pain,^1^ supplemented by other diagnoses agreed between two primary care physicians. Intra-abdominal lymphoma was omitted because codes do not report the anatomical site reliably. Gastro-oesophageal reflux disease, gastritis, oesophagitis, gastric/duodenal ulcer, and hiatus hernia comprised the composite outcome of ‘upper GI conditions’ because these conditions can overlap and precise anatomopathological diagnoses are rarely made in primary care.

Pre-specified diagnostic codes in the year before the index date were also identified, to quantify the number of disease-free participants on the index date.

### Outcome

The outcome was 1-year cumulative incidence (%) of pre-specified medical diagnoses in the year after newly recorded abdominal pain:

$$=\frac{\text{Number}\;\text{of}\;\text{new}\;\text{diagnoses}\text{in}1\;\text{year}\;\text{after}\;\text{index}\;\text{date}}{\text{Number}\;\text{disease}\;\text{free}\;\text{on}\;\text{index}\;\text{date}}$$

The incident diagnosis was determined by the first diagnostic code recorded in the year after the index date, where none of the codes for that diagnosis had been recorded in the previous year. The 1-year cumulative incidence rate for the incident diagnosis is reported with 95% confidence intervals (CIs) for males and females in age bands 40–59, 60–69 and ≥70 years. Data analysis was conducted using Stata (version 17).

### Missing data and bias

All code lists used in this study are available on request from the authors. Following standard practice, the absence of a code for a clinical event was interpreted as its non-occurrence.^17^ Confounding by sex and age were controlled by stratified analyses.

### Public and patient involvement

Patient and public involvement came from discussions from the public advisory group (of which the fourth author is a member) to the Cancer Awareness, Screening and Early Diagnosis Policy Research Unit, who felt that it was important to understand why GPs referred some patients for hospital investigations but not others when presenting with abdominal pain.

## RESULTS

**Figure 1.**
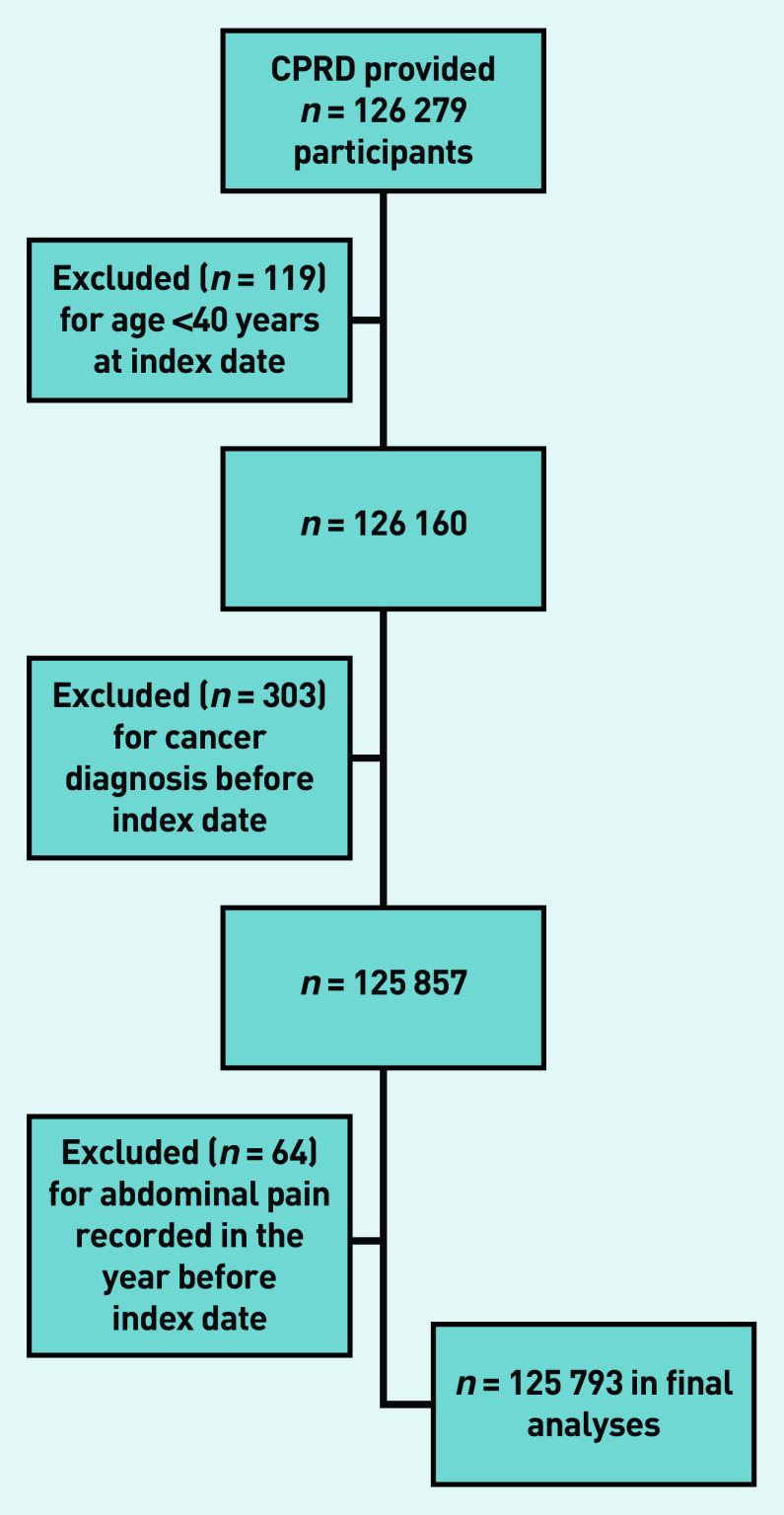
*Application of exclusion criteria. CPRD = Clinical Practice Research Datalink.*

The CPRD provided details of 126 279 potentially eligible participants, of whom 486 were excluded (Figure 1), leaving 125 793 in the study (Table 1).

**Table 1.** Sample characteristics

**Age group, years**	**Participants, *n* (% of total)**	**Male, *n* (% in age group)**
**40–59**	59 864 (47.6)	29 944 (50.0)
**60–69**	29 461 (23.4)	14 506 (49.2)
**≥70**	36 468 (29.0)	13 460 (36.9)
**Total**	125 793 (100)	57 910 (46.0)

The 1-year cumulative incidence values for males and females across the age bands, by diagnosis, are reported in Supplementary Table S3. Here, the focus is on diagnoses with a value of ≥1%.

### Diagnoses in males and females aged 40–59 years

At least one condition was diagnosed in 4952/29 944 (16.5%) males and in 6489/29 920 (21.7%) females, leaving 83.5% of males and 78.3% of females with unexplained abdominal pain.

In males, five conditions had a 1-year cumulative incidence of ≥1%, and all were non-malignant (listed in rank order):

- the group of upper GI problems (namely gastro-oesophageal reflux disease, hiatus hernia, gastritis, oesophagitis, or gastric/duodenal ulcer) (4.9%, 95% CI = 4.6 to 5.1);
- diverticular disease (2.3%, 95% CI = 2.1 to 2.4);
- irritable bowel syndrome (2.1%, 95% CI = 1.9 to 2.3);
- gallstones (1.4%, 95% CI = 1.3 to 1.6); and
- urinary tract infection (1.1%, 95% CI = 1.0 to 1.2).

The same five conditions also had a 1-year cumulative incidence >1% in females, but in different rank order:

- urinary tract infection (5.1%, 95% CI = 4.8 to 5.3);
- upper GI problems (4.0%, 95% CI = 3.8 to 4.2);
- irritable bowel syndrome (2.9%, 95% CI = 2.7 to 3.1);
- gallstones (1.4%, 95% CI = 1.3 to 1.6); and
- diverticular disease (1.5%, 95% CI = 1.4 to 1.6).

Additional diagnoses were uterine fibroids (1.3%, 95% CI = 1.2 to 1.4) and ovarian cysts (1.2%, 95% CI = 1.1 to 1.3).

### Diagnoses in males and females aged 60–69 and ≥70 years

At least one condition was diagnosed in 3278/14 506 (22.6%) males and in 3842/14 955 (25.7%) females aged 60–69 years. In the ≥70 years age group, the figures were 3386/13 460 (25.2%) males and 6180/23 008 (26.9%) females. No codes for the pre-specified diagnoses were recorded for 77.4% of males and 74.3% of females aged 60–69 years, or for 74.8% of males and 73.1% of females aged ≥70 years. Table 2 reports the cumulative 1-year incidence in rank order by sex and age group.

**Table 2.** Diagnoses with a 1-year cumulative incidence of ≥1% in males (*n* = 14 506) and females (*n* = 14 955) aged 60–69 years, and in males (*n* = 13 460) and females (*n* = 23 008) aged ≥70 years after newly recorded abdominal pain

**Sex, age group, and diagnosis**	**Number in 1 year**		**At risk, *n***	**Cumulative 1-year incidence, % (95% CI)**

	**After index date**	**Before index date**		
**Male, 60–69 years**				
Upper GI problems	808	639	13 867	5.8 (5.4 to 6.2)
Diverticular disease	602	241	14 265	4.2 (3.9 to 4.6)
Gallstone	336	70	14 436	2.3 (2.1 to 2.6)
Prostatitis	316	391	14 115	2.2 (2.0 to 2.5)
UTI	233	313	14 193	1.6 (1.4 to 1.9)
IBS	194	135	14 371	1.3 (1.2 to 1.6)

**Female, 60–69 years**				
UTI	856	1292	13 663	6.3 (5.9 to 6.7)
Upper GI problems	768	796	14 159	5.4 (5.1 to 5.8)
Diverticular disease	697	327	14 628	4.8 (4.4 to 5.1)
Gallstone	467	132	14 823	3.2 (2.9 to 3.4)
IBS	355	254	14 701	2.4 (2.2 to 2.7)

**Male,≥70 years**				
Upper GI problems	752	605	12 855	5.8 (5.5 to 6.3)
Diverticular disease	551	331	13 129	4.2 (3.9 to 4.6)
UTI	425	583	12 877	3.3 (3.0 to 3.6)
Gallstone	320	83	13 377	2.4 (2.1 to 2.7)
Prostatitis	317	482	12 978	2.4 (2.2 to 2.7)
Colorectal cancer	190	190	13 460	1.4 (1.2 to 1.6)
Cholecystitis	189	57	13 403	1.4 (1.2 to 1.6)

**Female,≥70 years**				
UTI	1608	2894	20 114	8.0 (7.6 to 8.4)
Diverticular disease	1351	790	22 218	6.1 (5.8 to 6.4)
Upper GI problems	1135	1147	21 861	5.2 (4.9 to 5.5)
Gallstone	560	194	22 814	2.5 (2.3 to 2.7)
IBS	299	291	22 717	1.3 (1.2 to 1.5)
Gastroenteritis	247	321	22 687	1.1 (1.0 to 1.2)

a *Diagnoses listed in order of descending 1-year cumulative incidence, by sex and age group. Upper GI problems include gastro-oesophageal reflux disease, hiatus hernia, gastritis, oesophagitis, and gastric/duodenal ulcer. GI = gastrointestinal. IBS = irritable bowel syndrome. UTI = urinary tract infection.*

Upper GI problems followed by diverticular disease were the most likely diagnoses in males aged 60–69 and ≥70 years. Females were most likely to be diagnosed with urinary tract infection, followed by upper GI problems or diverticular disease. Other conditions included gallstones and irritable bowel syndrome, which had higher 1-year cumulative incidence values in females than in males aged 60–69 years. For males, the 1-year cumulative incidence of prostatitis was similar to that of gallstones in age groups 60–69 and ≥70 years.

### Patterns with age

**Figure 2.**
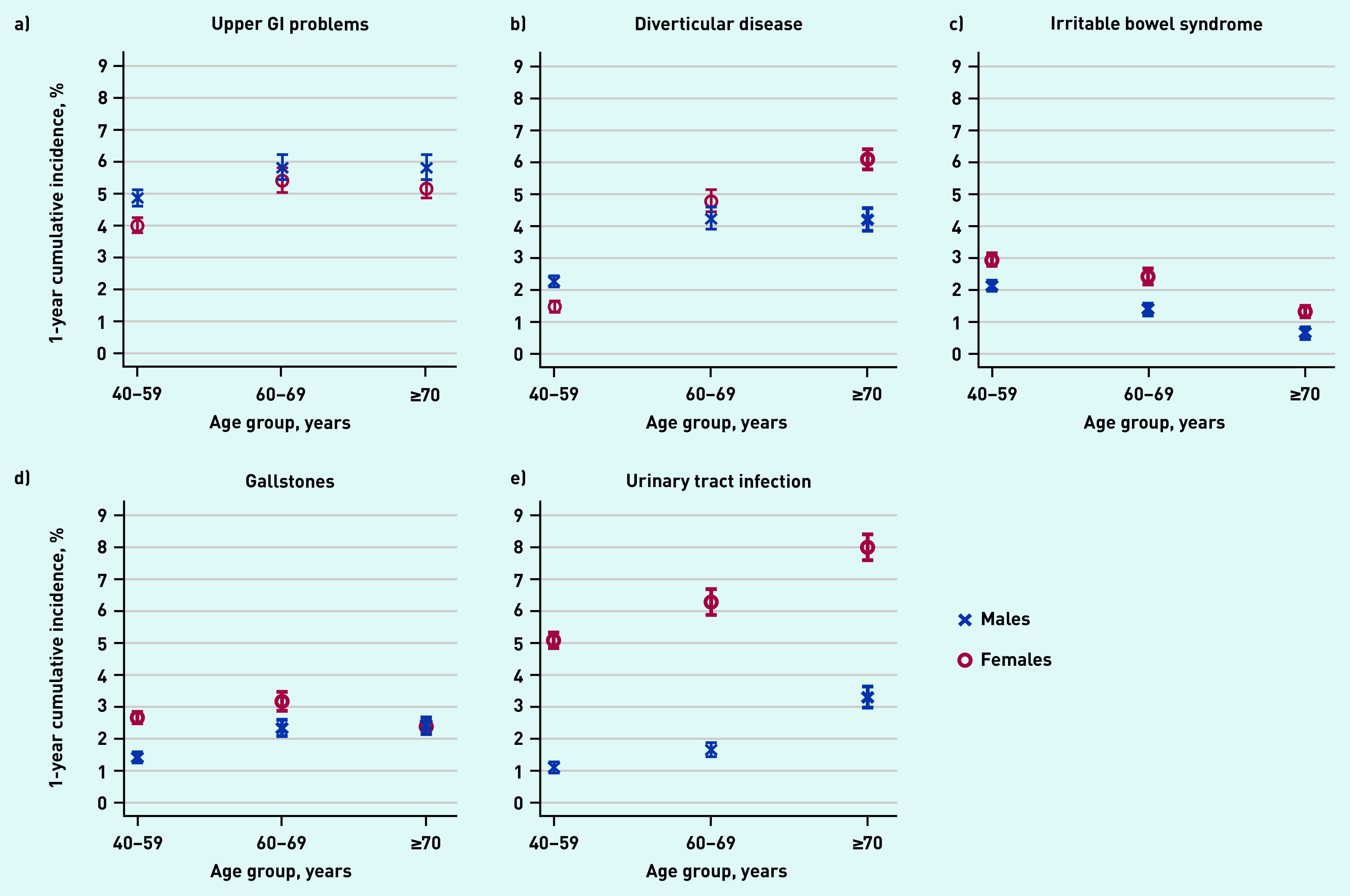
*The 1-year cumulative incidence (95% confidence interval) of the most common pre-specified diagnoses in males (blue crosses) and females (red circles) with newly reported abdominal pain, by age group. a) Upper GI problems (includes gastro-oesophageal reflux disease, hiatus hernia, gastritis, oesophagitis, and gastric/duodenal ulcer); b) diverticular disease; c) irritable bowel syndrome; d) gallstones; and e) urinary tract infection. GI = gastrointestinal*

The 1-year cumulative incidences of the four most common conditions in both sexes — upper GI problems, diverticular disease, gallstones, and urinary tract infection — were higher in the age group 60–69 years compared with 40–59 years (see Supplementary Table S3). The 1-year cumulative incidence increased further for the ≥70 years age group only for urinary tract infection, and for diverticular disease in females (Figure 2). In contrast, the 1-year cumulative incidence of irritable bowel syndrome decreased with age for males and females, remaining consistently higher in females. In males aged ≥70 years, it was <1% (0.6%, 95% CI = 0.5 to 0.8).

## DISCUSSION

### Summary

This study focuses on patients with abdominal pain, a common clinical scenario in primary care. The 1-year cumulative incidence (%) for a wide range of diagnoses that may present with abdominal pain for males and females aged 40–59, 60–69, and ≥70 years are reported. The four conditions most frequently diagnosed in males and females across the age groups were non-malignant: upper GI problems (that is gastro-oesophageal reflux disease, hiatus hernia, gastritis, oesophagitis, and gastric/duodenal ulcer), diverticular disease, gallstones, and urinary tract infection.

For males, upper GI problems and diverticular disease were the most likely diagnoses at all ages, and the 1-year cumulative incidence stabilised over the age of 60 years. For females, urinary tract infection was by far the most likely diagnosis, and the 1-year cumulative incidence increased across the age groups. Irritable bowel syndrome was also common, less so with increasing age and for males. Notably, >70% of participants in all age groups did not have a pre-specified diagnostic code in the year after their index date, suggesting that their abdominal pain remained unexplained.

### Strengths and limitations

This study used CPRD GOLD data, generally considered to hold data on a representative sample of patients consulting primary care.^17^ The code lists used in this study were comprehensive, developed with GPs using robust methods.^18^ To maximise the chances of identifying participants with newly recorded abdominal pain, individuals with an abdominal pain code recorded in the year before their index date were excluded. The well-reported limitations of using observational CPRD data include missing clinical information recorded solely in text fields.^19^ Text-only recording of abdominal pain may have led to up to one-third of possible inclusions being overlooked. Reassuringly, there is no evidence that the GP’s method of recording (coded versus free text) abdominal pain is associated with a subsequent diagnosis of cancer, but this has not been checked for non-malignant diseases.^19^ Despite this, the sample sizes were sufficient to report estimates with the required precision. Text-only recording of subsequent diagnoses is also possible, although no pertinent studies quantifying this were found.

To improve identification of patients with a cancer diagnosis, linked Cancer Registry data were used. Many of the non-malignant conditions investigated in this study are managed in primary care and do not require mandatory reporting to disease registries. This limited opportunities for validation through linked datasets. It was reassuring that, overall for 183 different diagnoses, a median of 89% of cases in the CPRD were confirmed (most frequently by contacting the GP) using codes.^20^ This suggests that any underestimation of 1-year cumulative incidence values will be small.

In this study, the choice of reporting 1-year cumulative incidence was deliberate, to inform GPs of the absolute risk of each pre-specified condition in patients with newly recorded abdominal pain within 1 year. This approach was preferred to use of positive predictive value because it could not be assumed that the prevalence of each pre-specified condition in this sample represented that of the general population. Also, omitting patients without abdominal pain meant this study could not determine the increased risk of conditions posed by abdominal pain, but one study reported incidence rate ratio of 6.2% (95% CI = 5.4 to 7.1) overall.^21^ For context, intra-abdominal cancer risk was nearly fourfold higher than baseline risk in males aged ≥70 years with abdominal pain,^16^ and baseline urinary tract infection incidence rates (per 100 person–years at risk) in males and females aged 75–84 years were 6.13 (95% CI = 5.25 to 7.00) and 14.34 (95% CI = 13.13 to 15.54), respectively.^22^ Finally, the present study did not identify other possible symptoms of the pre-specified non-cancer conditions, again because the research focus was abdominal pain.

### Comparison with existing literature

In a systematic review and meta-analysis of patients reporting abdominal pain to primary care, the underlying cause could not be specified for 12.7% to 63.8% of patients.^1^ This wide prediction interval reflects the heterogeneity of studies included. Nevertheless, the findings of the present study are inconsistent with this, with >70% of participants overall having no diagnostic codes after the index abdominal pain record. The are three possible explanations for this discrepancy. First, only 3 of the 14 studies included in the meta-analysis used clearly defined diagnostic categories. In contrast, the diagnoses in the present study were tightly defined with clinical input as being clinically relevant, and were identified using robustly compiled code lists that align closely with Read code or International Classification of Diseases (10th revision) code definitions of disease. The limited opportunities to validate these diagnoses using linked datasets in the present study is acknowledged. Second, the present study only included participants aged ≥40 years, whereas the meta-analysis included data from patients as young as 0–4 years. Third, the present study analysed coded data, whereas the studies contributing to the meta-analyses may have had access to text-only records. The small effect that this may have had on the estimates in the present study has been discussed above.

The stratification of analyses by age and sex in the present study, and differences in disease categorisation and outcome measures complicate direct comparison of the estimates with the systematic review’s meta-analysis.^1^ Their outcome is the number of diagnoses reported as a percentage of patients with abdominal pain, which will differ from the 1-year cumulative incidence used in the present study, as patients with pre-existing diagnoses were excluded from the outcome and denominator for each disease studied. It is reassuring that the most common diagnoses in the present study are similar to those of Viniol *et al*.^1^ For example, they report that 5.3% of patients were diagnosed with a urological disease and 5.2% with gastritis, which are in the same order of magnitude as the estimates of 1-year cumulative incidence for urinary tract infection and upper GI problems in the present study. They reported that 3.0% were diagnosed with diverticular disease, which is a little lower than the 1-year cumulative incidence estimates in the present study. This is probably because diverticular disease incidence increases with age and in the present study patients aged <40 years were omitted. In contrast, they reported higher proportions of patients diagnosed with gastroenteritis and irritable bowel syndrome at all ages. Again, this may relate to the minimum age of 40 years in the present study, because the incidence of these diagnoses decreases with age.

### Implications for research and practice

In this study it was not possible to identify whether some non-malignant diagnoses were initial misdiagnosis of a cancer. Further mixed-methods research exploring safety netting for patients whose abdominal pain persists despite treatment for a non-malignant diagnosis is recommended.

The majority of pre-specified diagnoses require primary care tests of blood, urine, or faeces, the major exception being the group of upper GI problems. This study suggests that clinicians might need to consider endoscopy for males and females with unexplained abdominal pain, particularly if unresponsive to acid-suppression treatments. Colonoscopy may also be useful to rule out alternative diagnoses in suspected irritable bowel syndrome, especially in older patients. In females, urinary tract infection had consistently the highest incidence across all age groups. This diagnosis is usually made within primary care. Other imaging modalities that may be indicated included: ultrasound for diverticular disease, gallstones, cholecystitis, or hernia; or computed tomography for diverticular disease, gallstones, or hernia. Having identified the conditions most commonly diagnosed following newly recorded abdominal pain, further research should seek to identify the additional predictive value of additional possible symptoms of these conditions.
